# A novel fluorescent marking clip for laparoscopic surgery of colorectal cancer: A case report

**DOI:** 10.1016/j.ijscr.2019.10.024

**Published:** 2019-10-17

**Authors:** Satoshi Narihiro, Masashi Yoshida, Hironori Ohdaira, Takayuki Sato, Daisuke Suto, Sojun Hoshimoto, Norihiko Suzuki, Rui Marukuchi, Teppei Kamada, Hideyuki Takeuchi, Yutaka Suzuki

**Affiliations:** a Department of Surgery, International University of Health and Welfare Hospital, 537-3, Iguchi, Nasushiobara City, Tochigi, 329-2763, Japan; b Center for Photodynamic Medicine, Kochi University, Kohasu Oko-cho 185-1, Nankoku, Kochi, 783-8505, Japan; c Department of internal medicine, International University of Health and Welfare Hospital, 537-3, Iguchi, Nasushiobara City, Tochigi, 329-2763, Japan

**Keywords:** Colorectal cancer, Fluorescent clip, Tattoo marking, VISION SENSE

## Abstract

**Introduction:**

In laparoscopic surgery, marking of tumor location has been gaining importance. Tattoo marking has been often used but the technique carries the risk of accidental peritoneal scattering or other organ injury. We have been involved in the development of a novel fluorescent clip for marking tumor sites and supposed that its usage would reduce risks related to other marking methods.

**Case presentation:**

A 52-year-old man was diagnosed with sigmoid colon cancer and polyp, and was scheduled for laparoscopic sigmoidectomy. On the day before operation, fluorescent clips (ZEOCLIP FS: Zeon Medical co, Ltd, Tokyo) were endoscopically placed around the tumor and polyp sites, 4 clips for each lesion attached every 90 degrees within the colonic lumen, respectively. During the operation, locations of the fluorescent clips were easily confirmed using a full-color fluorescent laparoscope, VISION SENSE (Medtronic Co., U.S.). Curative operation was performed accordingly with the preoperative pathological and radiological findings. The postoperative course was uneventful.

**Discussion:**

Locations of intraluminally placed fluorescent clips were clearly and easily recognized through the serosal layer of the intestinal wall using a fluorescent laparoscope. Complications related to dye scattering or intestinal wall/other organ perforation were not observed suggesting that future incorporation of this tumor site marking technique into laparoscopic surgery might be beneficial.

**Conclusion:**

The fluorescent marking clips were easily placed and recognized with a fluorescent laparoscope. This method is expected to be safe and risks of accidental puncture related to tattoo marking method can be reduced or almost eliminated.

## Highlights

• This is the first report on fluorescent marking clip for laparoscopic surgery.
• In laparoscopic surgery, marking of tumor location has been gaining importance.
• Tattoo marking technique carries the risk of accidental intestinal wall perforation leading to peritoneal scattering or other organ perforation.
• The fluorescent marking clips were easily placed and recognized with a fluorescent laparoscope.
• The fluorescent clip is expected to reduce risks related to other marking methods.

## 1 Introduction

**Fig. 1:**
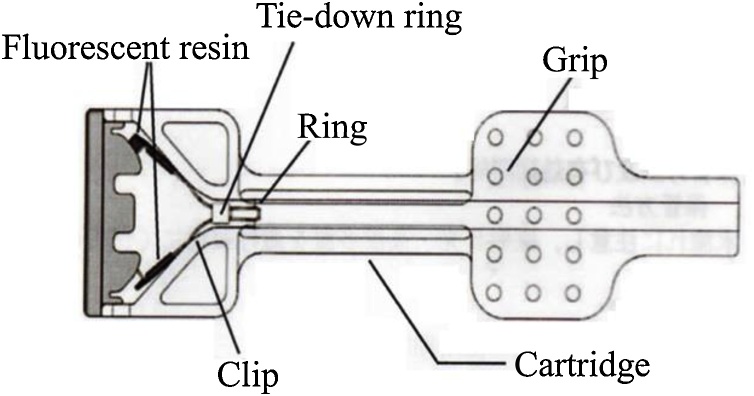
A schematic drawing of the fluorescent clip (ZEOCLIP FS) loaded into the cartridge. (Courtesy of Zeon Medical Co., Ltd., Tokyo).

Tumor site marking has become an increasingly important issue in colorectal laparoscopic surgery since the intestinal tract cannot be directly and tacitly examined within pneumoperitoneum. Even though it is possible to palpate the serosal side of the colon mucosa with laparoscopic devices (or directly, outside the abdominal cavity), some submucosal (SM) or muscularis propria (MP) lesions cannot be intraoperatively localized. In such cases, tattoo marking method has been often used but the procedure carries risks of accidental intestinal puncture potentially leading to peritoneal scattering or puncture(s) of other abdominal organs [[1], [2], [3], [4]]. We supposed that tumor marking with fluorescent dye-coated clips might provide a solution to the problems related to tattoo marking and requested development of a fluorescent marking clip to Zeon Medical Co., Ltd., Tokyo, Japan. The disposable Excitation ZEOCLIP FS clip with fluorescence wavelength emission peaks of 760 and 790 nm was manufactured (Fig. 1) and approved for clinical use (Registration No. 13B1X001111000020). We hypothesized that ZEOCLIP FS clips used with near-infrared radiation and fluorescent laparoscopic camera systems should be intraoperatively detected through the translucent intestinal wall on its serosal side even if it had been previously placed intraluminally. We present a case where the tumor marking fluorescent clips were used to localize colon cancer lesion during laparoscopic sigmoidectomy. This work has been reported in line with the SCARE criteria [5].

## 2 Case presentation

A 52-year-old man presented to our hospital with fecal occult blood. Based on endoscopy of the lower intestinal tract findings, he was diagnosed with sigmoid colon cancer (S type 2, 20 mm in size, cT2, cN0, cM0, stage I) and 6 mm-polyp, and was scheduled for laparoscopic sigmoidectomy with D3 lymph node dissection.

The patient’s consent on intraoperative use of the newly designed fluorescent clip and on reporting the study results had been obtained and the study was approved by the Institutional Review Board (No. 13-B-344).

**Fig. 2:**
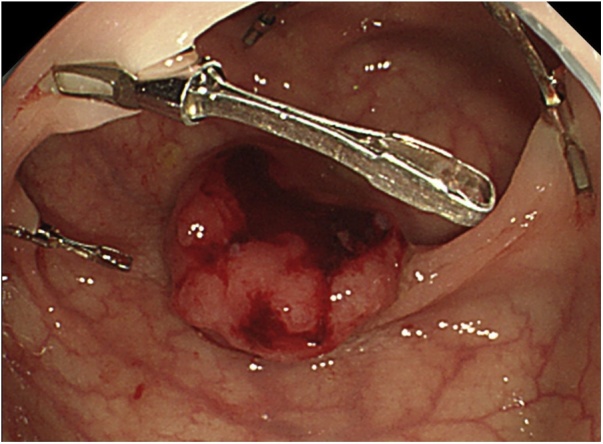
Fluorescent clips (ZEOCLIP FS: Zeon Medical Co., Ltd., Tokyo) placed endoscopically in 4 places every 90 degrees within the colonic lumen.

Following 2-days bowel preparation, fluorescent ZEOCLIP FS clips were placed during colonoscopy on the day preceding the surgery in two sites because removal of both the tumor and the polyp was planned. For each site, 4 clips were intraluminally placed every 90 degrees around the corresponding lesion, respectively (Fig. 2). Since the attachment of the first clip around the polyp had been insufficient, we added one more clip in the same site by clamping the intestinal wall with the clip during suction; this resulted in a total of 5 clips placed near the polyp lesion.

**Fig. 3:**
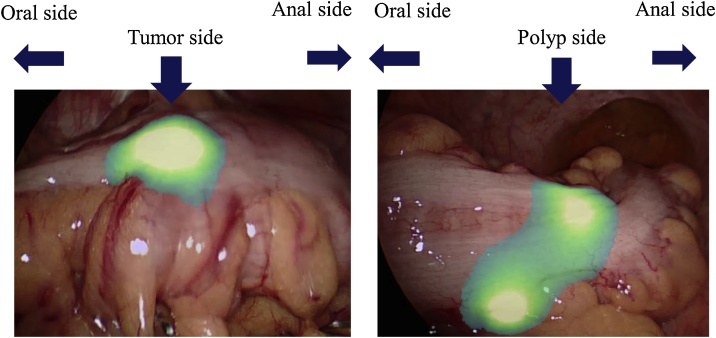
Intraoperative observation using a full-color fluorescent laparoscope VISION SENSE (Medtronic Co., U.S.) of the serosal layer.

**Fig. 4:**
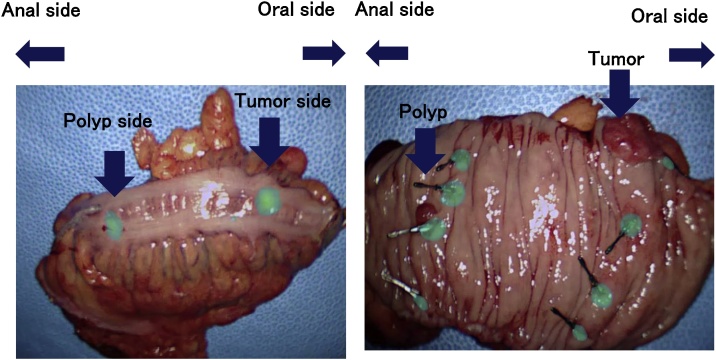
Resected specimen with clips marking the tumor and polyp locations.

The surgery was performed the following day as scheduled. Locations of the fluorescent clips were easily confirmed using a full-color fluorescent laparoscope, VISION SENSE (Medtronic Co., U.S.A.) (Fig. 3), and a sufficient margin was taken while separating the anal side of the tumor using an automatic suture device. The operation was performed without complications. Upon intraoperative examination of the dissected specimen, 4 clips were found in each placement location, i.e. of the tumor and of the polyp, respectively (Fig. 4). The insufficiently placed clip for marking of the polyp lesion site dropped. The curative operation was performed accordingly with the preoperative radiological, endoscopic and biopsy results. Pathological findings confirmed the preoperative findings (tumor: S type 2, 18 × 17 mm in size, pT1b, pN0, pM0; polyp: high grade tubular adenoma). The postoperative course was uneventful and the patient was discharged on the 7^th^ post-operative day.

## 3 Discussion

In the present case, we confirmed that intraluminally placed fluorescent clips could be easily detected through the translucent intestinal wall using a fluorescent laparoscope. The clips were easily placed preoperatively. Suctioning during sigmoidoscopy was applied to improve clip attachment and to avoid slipping by decreasing intraluminal pressure within the colon. According to manufacturer’s preclinical data, clip attachment weakens within a week after its placement though the fluorescence may be detected longer (data not shown). This is the first reported case on successful use of marking fluorescent clips to detect tumor site during laparoscopic surgery.

Conventional preoperative tumor site marking methods include tattoo marking, non-fluorescent clip placement and intraoperative endoscopy. Peritoneal scattering and accidental puncture of the intestinal wall and abdominal organs are potential drawbacks of the tattoo marking technique. Ink scattering may cause peritonitis or affect recognition of the tumor site and lead to inaccurate dissection margins [6,7]. This is particularly problematic in low rectal cancer cases when resection areas and reconstructions are often intraoperatively determined. In such cases, the tattoo marking also carries a higher risk of undesirable puncture. No definite rule exists on where and how much of black ink should be used for marking and there may be cases when the applied amount is excessive and interferes with accurate tumor localization. This may be further complicated by individual differences characteristic to the tumor site itself such as intestinal wall and surrounding fat tissue thickness [8].

Recently, there have been reports on applying indocyanine green (ICG) solutions instead of black ink for marking [9,10] however, the possibility of accidental puncture cannot be completely eliminated since the technique is basically the same as that of tattoo marking. Cases of peritoneal scattering with ICG were also reported [11].

Intraoperative endoscopy and non-fluorescent clipping have been introduced to mark and/or confirm tumor locations during surgery. Although intraoperative endoscopy may be useful, its disadvantages such as prolonged operation time, colon insufflation that interferes with operative procedure and need of a skilled endoscopist, might reduce its widespread application [12].

In our method of marking a tumor site with fluorescent clip(s), the clips were attached as with the conventional clip placement technique under endoscopy performed prior to surgery. The shape of the fluorescent ZEOCLIP FS clip did not significantly differ from those of non-fluorescent clips but due to fluorescent coating a slight decrease in grasping force might occur and that might cause potential slipping. More cases need to be examined to confirm this possibility.

## 4 Conclusion

This is the first case report on tumor site marking with fluorescent clips for laparoscopic tumor resection. Preoperative endoscopic placement of the fluorescent clips was easy as was their intraoperative recognition. The fluorescent clips might reduce possibility of accidental intestinal wall puncture that occasionally occurs with the tattoo marking technique.

## Declaration of Competing Interest

The authors declare no conflicts of interest.

## Source of funding

We have no sponsors.

## Ethical approval

This study was approved (approval N0.13-B-344) by the Research Ethics Committee at the International University of Health and Welfare, Tochigi, Japan.

## Consent

We had obtained such consent.

## Author contribution

SN have made substantial contributions to conception and design, or acquisition of data, or analysis and interpretation of data.

MY have been involved in drafting the manuscript or revising it critically for important intellectual content.

HO have get in on a discussion about this study.

DS have get in on a discussion about this study.

MK have get in on a discussion about this study.

YK have get in on a discussion about this study.

NS have get in on a discussion about this study.

SH have get in on a discussion about this study.

YS have given final approval of the version to be published.

All authors read and approved the final manuscript.

## Registration of research studies

This paper is case report.

## Guarantor

Satoshi Narihiro.

## Provenance and peer review

Not commissioned, externally peer-reviewed
